# Gout and Its Relations to Diseases of the Liver and Kidneys

**Published:** 1889-12

**Authors:** 


*Gout and its Relations to Diseases of the Liver and Kidneys.* By ROBSON ROOSE, M.D. Sixth Edition. London: H. K. LEWIS. 1889.

The rapid appearance of another edition of this book shows that it has been favourably received. We noticed the work in some of its previous issues, and we have now to record that some slight additions have been made as the result of further experience.

